# An Innovative Strategy for Sustainability Assurance in Orthopedic Surgery

**DOI:** 10.3390/medsci13030093

**Published:** 2025-07-22

**Authors:** Flaviu Moldovan, Liviu Moldovan

**Affiliations:** 1 Orthopedics—Traumatology Department, Faculty of Medicine, George Emil Palade University of Medicine, Pharmacy, Science, and Technology of Targu Mures, 540142 Targu Mures, Romania; 2 Faculty of Engineering and Information Technology, George Emil Palade University of Medicine, Pharmacy, Science, and Technology of Targu Mures, 540142 Targu Mures, Romania; liviu.moldovan@umfst.ro

**Keywords:** healthcare, sustainability, orthopedics, hospital, environment, alternative interventions

## Abstract

Background/Objectives: Due to the aging population, orthopedics has become the surgical field with the fastest and greatest growth, and health systems must therefore ensure the sustainable development of this field. The objective of this study is to identify the key areas and the most relevant activities for sustainability assurance in orthopedic surgery, as well as appropriate evaluation tools. Methods: Consisted in designing the key areas for sustainable surgery and evaluating them with the support of new indicators. These areas are based on the most current and relevant practices reported by hospitals around the world. This process was followed by practical validation in an orthopedic hospital. Results: Four key areas were revealed: rationalization and reduction of consumption, correct separation and recycling of waste, reuse of textiles and equipment, and anesthetics and alternative interventions. The indicators for the evaluation of these areas were developed with the support of results reported in international studies. Each indicator has five levels of fulfilment. Their importance is evaluated on a scale from 1 to 5. Conclusions: The practical implementation at an orthopedic emergency hospital highlighted the suitability of the indicators within the purpose of the research. Sustaining the values of these changes by surgeons and patients facilitates long-term results and the orientation towards assuring the sustainability of orthopedic surgery.

## 1. Introduction

Health systems contribute substantially to carbon emissions, with hospitals being primary contributors. Reducing these emissions through mitigation strategies has become a key priority in healthcare sustainability research. Recent global frameworks, such as those published by the World Health Organization (WHO), emphasize the urgent need for environmentally responsible practices in surgical settings [1]. In this context, requirements were formulated for the provision of net-zero medical assistance [2]. In Europe alone, an estimated 6 million tons of medical waste are generated annually, with operating theaters contributing up to one-third of this total [3]. Effective waste reduction measures in these high-impact areas are essential for lowering healthcare’s environmental footprint. Selective waste collection in the operating room is the first step in reducing environmental pollution and waste disposal costs. Hospital expenses can be reduced directly, by reducing energy consumption and waste volumes, and indirectly, by reducing the harmful effects of pollution on public health. This requires concerted efforts to improve environmental performance [4].

Anesthetists perceive the environmental impact of the anesthetic practices they use in the operating room and recognize the importance of their work in terms of environmental sustainability. However, they appreciate that there are barriers in the realization of ecological practices, due to the lack of protocols and guidelines for the implementation of ecological circuits, and the absence of waste separation bags or recycling containers [5], educational programs, and implementation strategies [6].

New evidence from systematic reviews confirms that sustainable practices in surgery, such as waste segregation, energy optimization, and reuse protocols, are feasible and impactful. A 2024 review by Keil et al. [7] highlights common strategies and measurement frameworks for carbon reduction across healthcare systems, while Lam et al. (2023) systematically categorized effective interventions for sustainable surgery [8]. Despite early foundational work in this field, current recommendations stress the integration of up-to-date environmental guidelines and clinical practice standards.

In orthopedics, interest in sustainability has intensified due to the rapid growth of surgical volumes associated with an aging population and increasing rates of trauma care. Organizations such as the American Orthopaedic Foot and Ankle Society and the EHS have identified orthopedic surgery—particularly arthroplasty—as a high-impact target for intervention [7]. Small-scale changes, including streamlined surgical trays and anesthetic alternatives, are now being supplemented with systems-level improvements that reduce waste and emissions without compromising patient safety [9]. A quarter to half of hospital waste is produced in the operating room, and its recycling in the perioperative setting is unusual, although there is a high potential for recycling [10]. Among the orthopedic specialties, arthroplasty has the greatest potential for recycling, being also the largest producer of waste [11]. Orthopedic surgeons must critically analyze the configurations required for surgical interventions, as minor changes in instrument trays and textiles used have a significant impact on the volumes of waste generated and greenhouse gas emissions [12,13]. The impacts of operating room activities, with high energy requirements and the generation of large volumes of waste, are accepted as necessary for the provision of quality care but have not been researched in detail [14]. Stacey et al. [15] shows the need to implement sustainable practices from an ecological point of view in operating rooms. By doing so, the healthcare sector contributes to a more environmentally sustainable society that does not jeopardize patient safety [16,17].

Another critical component of sustainable orthopedic practice is the use of digital and additive technologies. Computer-assisted surgical planning enables three-dimensional spatial modeling of anatomical structures, allowing for enhanced precision in surgical execution [18,19]. The use of 3D printing supports the creation of patient-specific implants, surgical guides, and anatomical models. These innovations reduce intraoperative material use and time, enhance outcomes, and align with sustainability objectives [20]. Furthermore, these technologies represent a paradigm shift in personalized care and resource efficiency [21].

In addition to technological innovations, alternative surgical and anesthetic interventions are gaining attention for their potential to reduce the environmental and material burden of care [22]. For instance, the WALANT technique (Wide Awake Local Anesthesia No Tourniquet) eliminates the need for general anesthesia and associated energy-intensive equipment, while maintaining high patient safety and satisfaction [23]. Similarly, minimal-access procedures and non-operative alternatives, where clinically appropriate, can reduce surgical time, waste generation, and sterilization demands. These interventions are particularly impactful in orthopedic subspecialties such as hand surgery and trauma care, where simplified techniques and lower resource dependency can translate into both ecological and economic benefits [24]. Recognizing their value, our study includes these strategies within the broader domain of sustainability-focused practice changes [25].

Due to the aging population, orthopedics has become the fastest growing and largest surgical field, requiring hospitals to allocate resources and advanced and critical care services in orthopedics [26]. Volumes of orthopedic surgeries are constantly increasing, also due to the increasingly frequent occurrence of disasters in which patients with injuries predominate, and the health system can be overwhelmed in such situations [27,28].

Research Question: What are the specific key areas, the best current practices, and measurable indicators that can reliably assess and guide sustainability assurance in orthopedic surgery?

Working Hypothesis: If sustainability in orthopedic surgery is structured into specific, evidence-based domains—such as resource rationalization, waste recycling, equipment reuse, and anesthetic alternatives—then these domains can be translated into practical performance indicators whose implementation can be assessed and improved over time.

Verification Strategy: This hypothesis will be tested through (1) a systematic review of global literature on sustainable surgical practices, (2) the design of core indicator sets aligned with these key domains, and (3) practical validation via an internal audit performed in a public orthopedic hospital using a structured evaluation matrix.

The objective of this study is to identify the key areas and the most relevant activities for the development of a sustainable healthcare system in orthopedic surgery, as well as appropriate evaluation tools.

## 2. Materials and Methods

### 2.1. Research Design

This quantitative, exploratory primary research was designed to investigate the research question posed in the Section 1. We have gathered, from scientific publications, the most recent and appropriate practices related to sustainable orthopedic surgery assurance that have been implemented and reported by international hospitals. The conduct of the study was approved by the Ethics Committee of the Targu Mures County Emergency Hospital (ECHTM). Gathering the data necessary for the research, the development of theoretical models and practical validation at the orthopedic hospital was carried out following the ethical principles of the Declaration of Helsinki.

### 2.2. The Key Domains for Sustainable Orthopedic Surgery

In establishing the key areas of sustainable orthopedic surgery assurance, we considered both authoritative clinical guidance and empirical evidence. The four selected domains: (1) rationalization and reduction of consumption, (2) proper separation and recycling of waste, (3) reuse of textiles and equipment, and (4) anesthetics and alternative interventions, were first identified in the guide on sustainability in the operating theater published by the Royal College of Surgeons of England [29]. To validate and refine this selection, we conducted a systematic review of international studies reporting on successful sustainability interventions in surgical settings.

These four domains were chosen based on their direct relevance to operating theater practices and their demonstrated potential for reducing the carbon footprint, waste volume, and environmental impact specific to orthopedic surgery. Importantly, they are operational areas where clinicians and hospital teams can implement changes autonomously, measure progress, and influence outcomes without requiring systemic restructuring.

While other areas, such as water management, transportation emissions, and broad staff education, are also important to hospital-wide sustainability, they were not included as core domains in our model because they either (a) cross-cut all surgical functions (e.g., staff education), (b) require infrastructure-level interventions (e.g., hospital-wide water systems), or (c) extend beyond the typical scope of decision-making within orthopedic departments (e.g., patient or supply chain transportation logistics). Nonetheless, some of these factors are embedded within our selected domains (e.g., water reduction through surgical hand disinfection protocols or energy conservation initiatives), and future extensions of the framework may expand to include them more explicitly.

### 2.3. Data Collection and Analysis

Our research continued with the collection, from the scientific literature, of the most recent successful practices related to sustainable orthopedic surgery assurance.

We explored the medical literature from the most representative databases, such as Medline (via Ovid), Embase, PubMed, Cochrane, Scopus, and Web of Science. The search was conducted according to the following keywords: sustainability or environment or carbon footprint AND orthopedics or surgery or interventions AND planetary health or climate change or minimize. We prioritized English language papers, and recent articles—mostly those from 5–10 years prior to the search date presenting new knowledge supported by evidence—were screened.

From here we extracted primary and secondary results of activities that support the design of sustainability assurance practices in orthopedic medical services, but also identified the barriers that do not support it. The information was used to design indicators in the four key areas.

### 2.4. Validation in Practice of the Developed Theoretical Model

The theoretical model was pilot tested with a team of orthopedic specialists and quality assurance personnel. They suggested some improvements that the authors implemented in the content of the evaluation tool for sustainability assurance in orthopedic surgery. The research continued in the experimental part with validation in practice of the developed theoretical model. For this, we tested the theoretical developments in the four key areas—rationalization and reduction of consumption, correct separation and recycling of waste, reuse of textiles and equipment, and anesthetics and alternative interventions—at the Emergency County Hospital Targu Mures (ECHTM), within the Orthopedics–Traumatology Department [30].

The human resource involved in the practical testing of the developed model was a team of auditors composed of four people who work within the hospital in the field of health or hospital quality assurance: the principal physician of the orthopedic department, a healthcare worker responsible for quality management, the head orthopedic assistant, and a resident orthopedic physician. The audit team was intentionally composed of four individuals with complementary roles and direct involvement in orthopedic care and quality assurance: the principal physician of the orthopedic department (clinician), the hospital’s quality management officer (administrator), the head orthopedic nurse (surgical staff), and a resident orthopedic physician (junior clinician). All auditors were internal staff members but were selected based on their multidisciplinary perspectives, familiarity with hospital workflows, and prior experience with quality audits. Their selection was based on their expertise, familiarity with hospital workflows, and prior involvement in quality improvement initiatives.

Prior to the assessment all auditors participated in a structured preparatory session led by the study authors. This session reviewed the content and scope of each indicator, provided standardized definitions for each indicator level, and offered real-world examples for each tier of performance. This session acted as a calibration exercise to align evaluators’ expectations and scoring behavior.

Although no formal statistical calibration (e.g., inter-rater reliability testing) was performed in this initial validation, a pilot assessment of the indicators was carried out using a small set of real-world examples. This served as a calibration exercise to align expectations and scoring behaviors. During the main audit, the team used a consensus-based scoring approach, discussing each indicator level until a shared agreement was reached to minimize variability and enhance internal validity. In future stages of the model’s validation, especially in multicenter settings, we plan to incorporate quantitative methods such as the Intraclass Correlation Coefficient (ICC) to assess inter-rater reliability, as well as conformity analysis to evaluate consistency across auditors. These additions will help refine the scoring system and ensure its robustness, reproducibility, and objectivity in broader applications.

## 3. Results

### 3.1. Indicator Contents and Evaluation Grids for the Key Areas

In continuation of the study, the 40 bibliographic references extracted from the scientific literature were used to develop the content of the indicators and the evaluation grids related to the four key areas, as presented below.

#### 3.1.1. The Rationalization and Reduction of Consumption Key Area

This area relates to the rationalization of material consumption and the reduction of energy consumption in the operating room. Wormer et al. [31] present streamlining initiatives whereby disposable foam pads were replaced by reusable gel pads. The switch from soap to an alcohol-based waterless scrub was also made. Electrical batteries from the operating room, which were previously thrown away, were redistributed to the hospital or donated to charity.

The reduction of energy consumption was realized using the “Power Down” initiative, through which all the lights are turned off and all equipment that is not used for anesthesia is also turned off. Energy consumption can be reduced by turning off operating theater lights and workstations when not in use, switching to LED and motion sensor lighting. Surgical handwashing can be replaced with alcohol-based products to reduce water consumption [32].

An excellent opportunity to save costs by cutting the volume of reusable waste in the operating room is the use of reduced surgical trays. On them are placed instruments that are used more than 50%. This has the effect of lowering costs by 20% per surgical tray. However, it requires a multidisciplinary approach [33]. Thiel et al. [34] show that surgical waste and expenses can be lowered by minimizing materials brought into the operating room in single-use packages. They recommend the use of the “minimal” personalized package of single-use surgical supplies. To this is added the size of the package as a determining factor in the generation of waste. Surgeons must agree on the minimum number of instruments required for each of the procedure trays [35]. Designed to reduce the number of unused or redundant instruments in total joint arthroplasty, Lean methodology has the potential to improve surgical efficiency and generate substantial cost savings [36]. The surplus of instruments creates costs in the operating room, requiring an optimization of surgical supplies, as shown by Helmkamp et al. [37]. A new disposable supply kit was formulated based on the recommendations of Velicki et al. [38], who analyzed the minimum elements required for carpal tunnel release and trigger finger release.

In Steam Sterilization and Packaging of Reusable Surgical Instruments, Rizan et al. [39] show that the carbon footprint of decontamination and packaging tools was lowest when tools were part of kits (66–77 g CO2e per tool), with a two- to threefold increase when tools were individually packed (189 g CO2e per instrument). The surgical principles of “minor field sterility” and Wide Awake Local Anesthesia No Tourniquet (WALANT), reduce surgical waste and surgical costs without affecting patient safety or patient satisfaction [40].

Further streamlining is possible in the sterilization and handling of surgical equipment by replacing the ubiquitous polypropylene plastic sheeting with metal casings and reintroducing reusable surgical gowns. In orthopedics, consumption can be reduced by successfully reprocessing and reusing external fixators, razors, blades, burs, and tourniquets [41,42].

With the support of these practices, the rationalization and reduction of consumption indicator was elaborated (Table A1).

#### 3.1.2. The Proper Separation and Recycling of Waste Key Area

Health workers have limited awareness and experience in medical waste management, due to the inadequate implementation of national guidelines. In the study by Letho et al. [43], only 61.3% of investigated medical facilities correctly separated waste according to national guidelines. In the study [44], 56.4% of the participants had good waste separation practices. Continuous enforcement of waste management rules and regulations, by providing training and instructional posters in the work area, and making color coded bins available, is important.

To improve waste segregation practices, health authorities need to focus on the sufficient allocation of waste containers on site. In addition, regular training on standard procedures that improve adherence to segregation practices is needed [45]. Pindi et al. [46] proposed a simple scoring system, which is used to improve biomedical waste segregation compliance. In this system, at the end of each month, area-wide compliance is easily assessed, and areas with low scores are trained. Identifying and correcting errors in waste separation is possible by using Nightingale charts within a management information system [47].

Traditionally, the perfusion bypass circuit is emptied by the cardioplegia pump starting shortly after decannulation and heparin reversal. By adding 2000 mL priming solution, the bypass circuit is completely flushed and clear. Diverting the bypass to bag waste results in reduced environmental impact and annual cost savings. This process not only releases significantly less carbon dioxide into the environment, but also helps generate renewable energy [48].

Infusion circuits are mainly contaminated with polyvinyl chloride and polycarbonate and are difficult to sterilize, reuse, or recycle. Sterilizing, dechlorinating, and recycling the infusion circuit is a promising way to reduce the ecological impact of infusion waste [49].

Effective waste management of biomedical materials is facilitated by recycling and recovery. Proper waste separation and recycling protocols should be strictly followed to eliminate the risk of contamination. However, in the selection of reuse methods, the preservation of the functionality and original properties of the materials must be taken into account [50]. Among the orthopedic procedures, arthroplasties are one of the largest producers of waste and have the highest material recycling potential of up to three quarters [9].

With the support of these practices, the correct separation and recycling of waste indicator was elaborated (Table A2).

#### 3.1.3. The Reuse of Textiles and Equipment Key Area

Van Straten et al. [51] demonstrated five times lower costs and less impact on climate change when reprocessing and reusing disposable face masks. Life cycle assessment of airways from single-use and 40-time reusable laryngeal masks revealed a more favorable environmental profile for reusable masks. The sources of impact for disposable masks are polymer production, packaging, and waste management. For reusable masks, the sources of impact are washing and sterilization [52]. Vozzola et al. [53] conducted a comparative study of the life cycle activities for reusable and disposable surgical gowns. They show that reusable gowns reduce solid waste generation (84%), greenhouse gas emissions (66%), blue water consumption (83%), and energy consumption from natural resources (64%).

The pulse oximeter is intensive medical equipment globally used in emergency care. Reusable pulse oximeters generated fewer greenhouse gas emissions per day than their disposable counterparts [54]. In an Australian hospital with six operating theaters, replacing reusable anesthetic equipment with single-use variants increased CO_2_ emissions by almost 10%. They observed that water consumption is higher for reusable equipment [55]. Chauvet et al. [56] compared currently used disposable and reusable laparoscopic medical devices in terms of safety, cost, and carbon footprint. The study concludes that reusable medical instruments, especially trocars, present appreciable advantages from a medical and economic point of view. Anesthesiologists use laryngoscopes in the operating room, which can be reusable or disposable. Sherman et al. [57] evaluated the purchase options of the devices. The reusable ones presented a considerable cost advantage. They offer a better environmental option by avoiding excessive cleaning of reusable laryngoscope handles and blades. Reusable central venous catheter insertion kits are less expensive than disposable kits. However, efforts to minimize the environmental footprint of these items must be aimed at reducing water consumption and energy used for cleaning and sterilization. The relative environmental effects of reusable items are significantly altered depending on the source of electricity in the hospital [58]. For lumbar fusion surgeries, single-level reusable and disposable instruments can be used. A comparative life cycle assessment reveals that the cleaning and sterilization process selected for reusable instruments is responsible for up to 90% of greenhouse gas emissions. It decides which system is advantageous from an environmental perspective. The future development of surgical instruments from an environmental perspective should reduce the number of items that must be cleaned and sterilized for a surgical intervention [59].

The impact on the environment of hybrid instruments containing reusable and disposable components used in the laparoscopic treatment of cholecystectomy compared to disposable instruments is almost four times [60]. Shifting from single-use products in robotic surgery to hybrid equivalents that are mainly reusable but with a small disposable component improves the environmental impact. The use of 5 mm hybrid ports compatible with emerging robotic systems lowers the carbon footprint by 83%, compared to the use of single-use equivalents [61,62]. The increased environmental impact of minimally invasive surgery may not sufficiently offset the clinical benefit. Alternative surgical approaches using reusable equipment, repackaging, and increasing staff awareness of open and unused equipment and avoiding desflurane, reduce greenhouse gas emissions and waste [63].

By reprocessing intermittent pneumatic compression sleeves, the carbon footprint is reduced by 40% compared to single-use devices [64]. The ecological footprint is also significantly influenced by the packaging of medical devices. Each medical device should possess comprehensible ecological key numbers to decrease the cost of the items [65].

With the support of these practices, the reuse of textiles and equipment indicator was elaborated (Table A3).

#### 3.1.4. The Anesthetics and Alternative Interventions Key Area

The reduction of respiratory effects, carcinogenic impact, and non-cancerous impact is facilitated by the integration of biopolymers in the construction of medical products. However, the manufacture of biopolymers is associated with a high impact on the environment, due to agricultural inputs [66].

Ciprofol has high efficacy, good selectivity, and fewer side effects, indicating good potential for clinical application [67].

In the management of rhegmatogenous retinal detachment, reduction of carbon emissions can be achieved by using air buffer instead of fluorinated gases [68].

Sevoflurane or propofol are used for general anesthesia. An assessment of anesthetic approaches reveals similar carbon footprints. An implicit low-carbon approach is spinal anesthesia. Other alternatives consist of the use of low-flow anesthesia/total intravenous anesthesia [69].

With the support of these practices, the alternative interventions and anesthesia indicator was elaborated (Table A4).

The indicator levels presented in Table A1, Table A2, Table A3 and Table A4 are cumulative rather than mutually exclusive. This means that achieving a higher level of an indicator assumes that the criteria outlined in all preceding levels have also been fulfilled. This hierarchical structure reflects a progressive implementation pathway and aligns with the principles of continuous quality improvement. It allows healthcare institutions to assess not only the presence of individual sustainability measures but also the depth and integration of their implementation.

#### 3.1.5. Levels of Importance for Indicators

In general, the designed indicators show different levels of importance for healthcare facilities. For this reason, we introduced a second variable that quantifies this aspect. Its evaluation is carried out on five levels:

1. Unimportant: the subject is of little importance and does not affect the achievement of the organizational sustainability objectives;
2. Low importance: failure to comply with the requirements could slightly affect the achievement of the sustainability objectives;
3. Important: failure to comply with this requirement could adversely affect achievement of the sustainability objectives;
4. Very important: failure to comply with the requirement could compromise achievement of the sustainability objectives;
5. High importance: failure to comply with the requirement may even compromise achievement of the sustainability objectives and sustainable development.

The placement in these levels is based on the auditors’ assessment, supported by criteria such as the content of the organizational objectives related to sustainability, the quantification method and the degree of their achievement, the potential to improve environmental sustainability, the potential to reduce CO_2_ emissions, the number of non-conformities registered in the former audit, the progress in the implementation of corrections and corrective actions, etc.

### 3.2. Indicator Matrix and Continuous Improvement Cycle

We developed the matrix of indicators (Table 1), which provides an overview of the indicators designed for the evaluation of sustainable orthopedic surgery and their levels.

**Figure 1:**
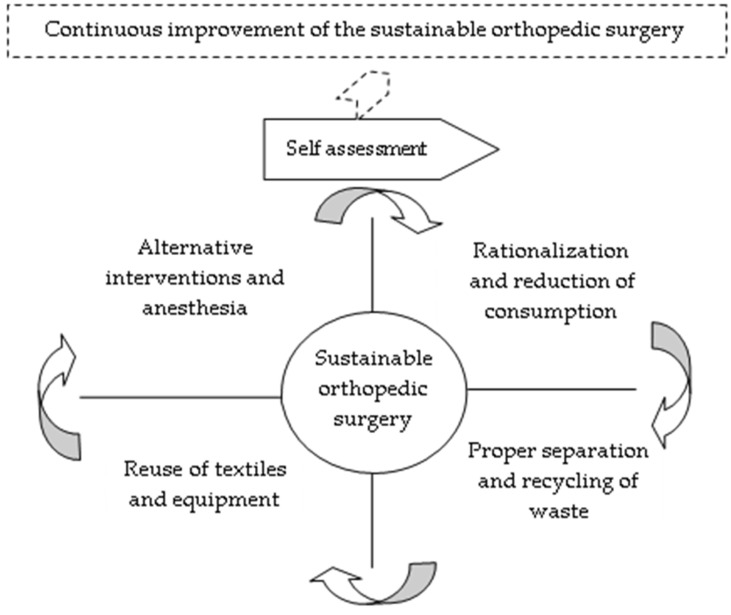
Continuous improvement cycle of the sustainable orthopedic surgery assurance.

We designed the theoretical model for evaluating the degree of innovation and sustainability in an orthopedic healthcare facility with the support of Deming’s continuous improvement cycle (Figure 1).

It summarizes the four key areas of sustainable orthopedic surgery, followed in the plan-do-check-act sequence: rationalization and reduction of consumption, proper separation and recycling of waste, reuse of textiles and equipment, and alternative interventions and anesthesia. These areas are represented in the continuous line boxes adjacent to the sustainable orthopedics assessment process. Their progress at each evaluation cycle is quantified through the five levels of fulfillment of the indicators that are represented externally by callouts with broken lines.

**Figure 2:**
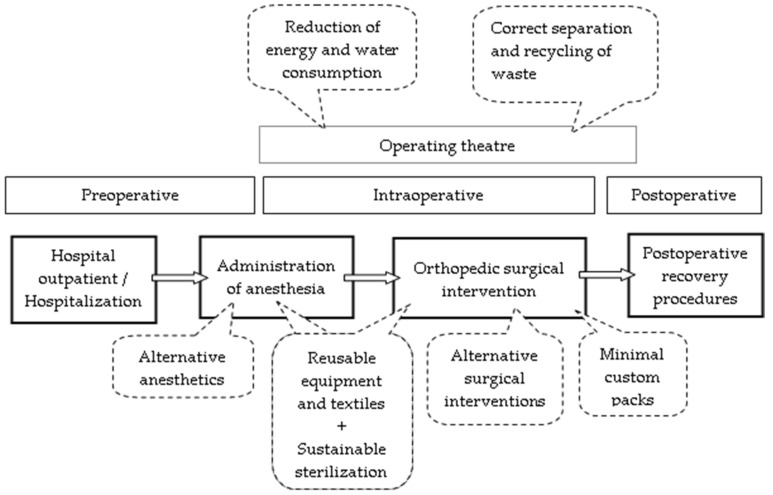
The positioning of the intervention areas of sustainable orthopedics surgery assurance in relation to the operative route.

Our study identified four key areas for sustainable orthopedic surgery. We found that these areas are positioned in all stages of the operating route, as can be seen in Figure 2.

The key areas reduction of energy and water consumption as well as correct separation and recycling of waste are positioned in all phases of the operating theater. The key areas alternative anesthetics, sustainable sterilization, and reusable equipment and textiles, are positioned in the phase of administration of anesthesia. The key areas alternative surgical interventions, minimal custom packs, sustainable sterilization, and reusable equipment and textiles are positioned in the phase of orthopedical surgical intervention.

### 3.3. Practical Validation at the Orthopedic Emergency Hospital

In the next step of the research, the assessment tool for sustainability assurance in orthopedic surgery is elaborated (Table 2). By comparing the qualitative description of the four indicators at ECHTM with the descriptions in Table A1, Table A2, Table A3 and Table A4, the auditors obtained the achievement levels (the “Level (Li)” column in Table 2). The values related to the tabular column “Importance (Ii)” were given by the auditors according to the specifications formulated inSection 3.1.5. Finally, the sustainability indicator (Si) is determined as the product between the level of achievement (Li) and the importance of the indicator (Ii).

It is worth noting that the structure of the assessment tool in Table 2 conceptually aligns with the Failure Modes and Effects Analysis (FMEA) framework commonly used in healthcare quality and risk management. Specifically, the Sustainability Indicator (Si) is calculated as the product of two core variables—the level of implementation (Li) and the importance (Ii) of each indicator—which parallels FMEA’s use of occurrence and severity scores. While our study does not apply a formal FMEA process, the methodological logic is consistent with prioritization models based on risk or impact-weighted scoring. By adapting FMEA principles to the context of sustainability in orthopedic surgery, our contribution lies in extending these established evaluation mechanics to a non-traditional domain, the environmental and resource performance, thus supporting structured decision-making in green healthcare practices.

**Figure 3:**
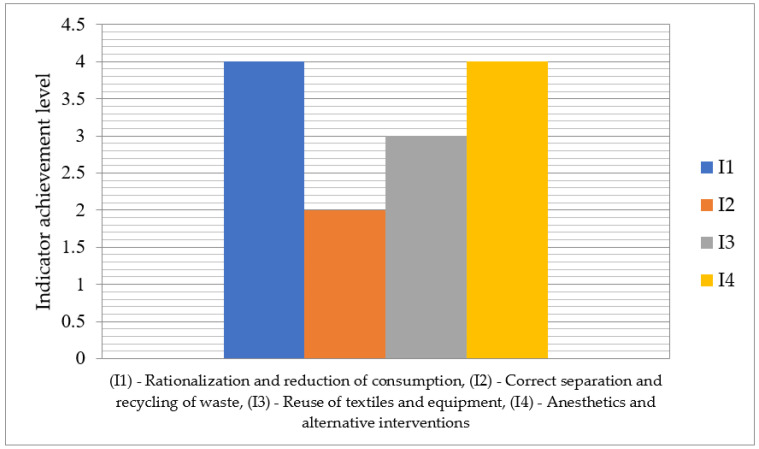
Achievement levels of indicators for the sustainability assurance in orthopedic surgery.

In Figure 3 it is depicted the achievement level of indicators related sustainable orthopedic surgery assurance on the scale 1–5. The indicator I2—Correct separation and recycling of waste has the lowest level 2, while the indicators I1—Rationalization and reduction of consumption and I4—Anesthetics and alternative interventions have the highest level 4, with no indicator reaching the maximum level 5.

**Figure 4:**
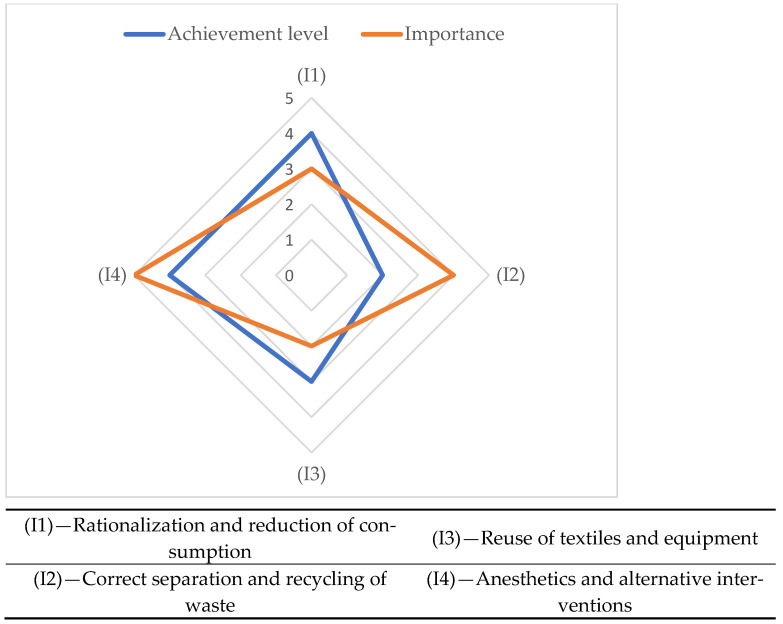
The sustainability assurance evaluation graph in orthopedic surgery.

The evaluation graph in Figure 4 shows the correlation between the achievement level and importance of the indicators related to sustainability assurance in orthopedic surgery.

By summing up the values of individual sustainable orthopedic surgery indicators in Table 2, the global sustainability indicator (GS_OSD_) is deduced [70] as follows:

(1) $${GS}_{OSD}=\sum_{i=1}^{4}S_{i}=\sum_{i=1}^{4}L_{i}\cdot I_{i}=46$$

By summing up the maximum values of the indicators, the maximum global sustainability value for sustainable orthopedic surgery (GSmax_OSD_) is obtained as follows:

(2) $${GSmax}_{OSD}=5\cdot \sum_{i=1}^{4}L_{i}=5\cdot 14=70$$

By reporting the current value of the global indicator to its maximum value, an image of the fulfillment degree of the requirements related to sustainable orthopedic surgery is obtained (FDR_SOD_) as follows:

(3) $${FDR}_{SOD}=\frac{{GS}_{OSD}}{{GSmax}_{OSD}}\cdot 100=\frac{46}{70}\cdot 100=65.71\%$$

**Figure 5:**
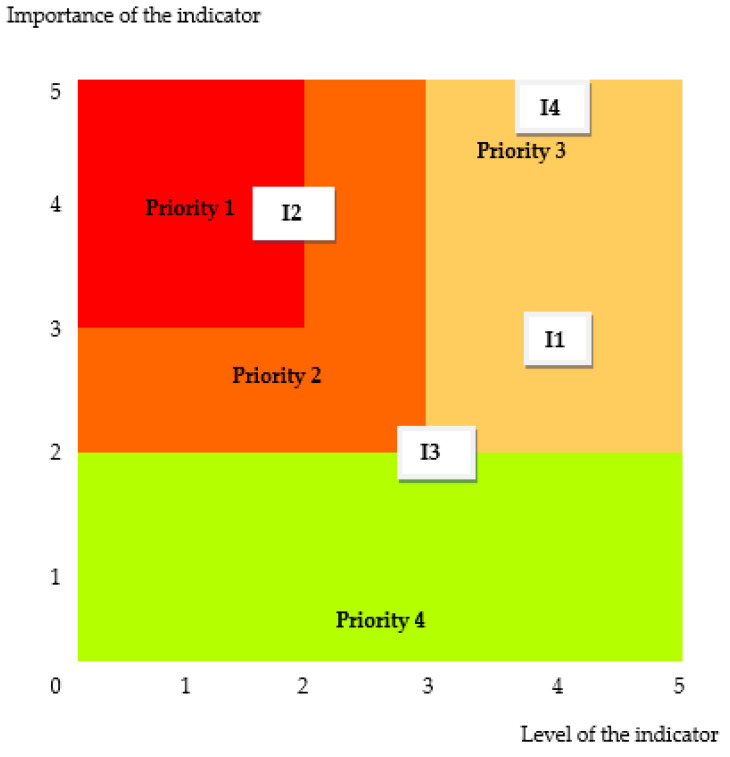
The prioritization framework for sustainability assurance in orthopedic surgery: (I1)—Rationalization and reduction of consumption, (I2)—Correct separation and recycling of waste, (I3)—Reuse of textiles and equipment, and (I4)—Anesthetics and alternative interventions.

The degree of fulfillment of the requirements related to sustainable orthopedic surgery reflects the extent to which they are fulfilled within the hospital where the evaluation took place. For its growth, it is necessary to adopt improvement measures. The priority of measures can be decided with the prioritization framework of Figure 5.

It is an Eisenhower matrix in which the importance and urgency of treating the indicators is given by framing between the extremes of high priority (1) and low priority (4). The highest priority must be given to the indicator (I2)—Correct separation and recycling of waste.

## 4. Discussion

In this study we found that the indicators designed for the evaluation of the four key areas are adequate for the purpose of the research. We appreciated that they correspond to the evaluation requirement of orthopedic surgery sustainable assurance. We discovered that they are compatible with other reference frameworks implemented in the hospital, namely the legal requirements for accreditation of health units with beds [71], or those in ambulatory care [72], and with the European framework for assessing the quality of DUQuE hospitals [73]. As regards the composition of the indicators, we found that they can still be adapted to the specifics of the health unit. For a good understanding of the levels that characterize them, a glossary of terminology can be developed. This facilitates mutual understanding between auditors and auditees.

Another finding is related to the human resources involved. Auditors must be well trained in the aspects related to the sustainability of medical activities. They must establish good communication relations with the auditees. The principal auditor must be a stately person and a good manager. Regarding sustainability, we found that the design methodology can orient the medical staff, and the entire hospital, towards sustainable development [74].

Another finding of the study shows that the indicator (I2)—Correct separation and recycling of waste must be treated with priority. In this way, sustainable orthopedic surgery can improve and contribute, in the short term, to developing the environmental sustainability of the hospital. For this, it is necessary to implement an effective tracking system for errors in waste segregation. The sterilization of the bypass infusion circuits must be carried out with additional priming treatments. New reuse methods that consider the preservation of functionality and original properties of materials must be implemented. Opportunities for recycling materials from hip and knee arthroplasties must be exploited.

In our study, we found that reusable equipment has a better environmental profile compared to single-use equipment. There are opinions, like MacGain [55,58], which show that CO_2_ emissions from sterilization processes depend on the electricity source of the hospital. This, in turn, can decide the ecological superiority of reusable equipment compared to disposable equipment. This requires analysis of the contextual factors that influence the effectiveness and complexity of changes in sustainable surgical practice. In addition, the health environment is constantly evolving, which requires the implementation of interventions in rhythm with potential benefits for the environment.

We also found that when the surgical team decides on the surgical technique or mode of anesthesia, sustainability is only one of the many factors considered. The decision-making elements that take precedence are the intervention requirements in the underlying pathology, the patient’s prognosis, the surgeon’s experience, the availability of resources, economic efficiency, and hospital and institutional specifics. Although some studies, such as Wormer et al. [31] or Rizan et al. [39], indicate the long-term financial benefits of sustainable interventions, in our study, we found limited evidence regarding the economic efficiency of sustainable surgical interventions. We believe that sustainable surgery is only effective if sustainability is balanced with cost-effectiveness, as well as other factors that go into surgical decision-making.

Although several studies, such as that of Eckelman et al. [52], present sustainable interventions used for more than a decade, in our study, we noted that they are not widely implemented in surgery departments. For this reason, the effectiveness of sustainable interventions should not be analyzed as detached from the context. Analysis of their theoretical effectiveness must be followed by how they can be widely implemented in real clinical practice. In addition, they must be acceptable to all stakeholders, surgical teams, managers, and patients. Thus, if the stakeholders support the value of the change, the designed interventions will be more appropriate, and their results will be long-lasting and more effective.

Although this study did not perform a formal economic analysis, several interventions included in the sustainability indicators have demonstrated favorable cost-effectiveness in previously published work. For example, reduction in surgical instrument tray size has been associated with up to 20% cost savings per procedure, primarily due to decreased sterilization load, maintenance, and material handling [33,34]. Similarly, replacing single-use foam pads with reusable gel pads or using alcohol-based hand rubs in place of surgical scrubs contributes not only to environmental goals but also to reduced procurement and utility costs [31,32]. Reprocessing of external fixators, blades, and tourniquets, when implemented with proper sterilization protocols, can yield meaningful long-term savings, particularly in high-volume orthopedic centers [37,39]. Reusable surgical gowns and drapes have also been shown to reduce solid waste by over 80% and offer life-cycle cost advantages compared to disposable alternatives, despite higher initial acquisition costs [53]. The shift towards hybrid surgical instruments in robotic surgery, which combine reusable and disposable components, demonstrates up to 83% reduction in carbon emissions and shows promise for cost optimization over time [61,62].

However, return on investment (ROI) varies depending on context, especially with regard to electricity pricing, labor costs, and procurement models. Furthermore, some sustainability improvements, such as information system integration or advanced sterilization infrastructure, may require initial capital investment. Therefore, future stages of our research will aim to include a structured cost-benefit analysis or life-cycle cost assessment of these interventions across different hospital settings. A key strength of the indicator system developed in this study is its compatibility with economic modeling. Each indicator can be paired with cost data to assess cost per sustainability point, or projected ROI based on baseline and post-intervention resource consumption metrics. This opens the door for future use of the tool as a decision-making framework for hospital administrators and policymakers balancing environmental and financial outcomes.

The limitations of this study refer primarily to the stage of exploring specialized literature. Although we used a robust search strategy, it is possible that, due to the dynamics of the field, not all available evidence was included in the study. It is possible that not all successful medical practices that could have contributed to a better design of the indicators have been identified. Furthermore, several selected studies were more cross-sectional, with retrospective data, than longitudinal. We appreciate that, in this way, we collected initial evidence, but, in the future, only longitudinal studies will provide a measure of intervention effectiveness. Another limitation comes from the validation setting, which was conducted in a single orthopedic emergency hospital with a specific public healthcare profile. While this allows in-depth pilot testing with experienced local auditors, it restricts the generalizability of findings to other hospital types, such as private clinics, general hospitals, or facilities in different healthcare systems. Additionally, the current study relied on a consensus-based evaluation method without applying formal inter-rater reliability testing. As such, inter-panel consistency and objectivity remain to be demonstrated. Future research directions require conducting additional experiments to address identified limitations, such as new database research, followed by expanding the content of indicators. Much of the literature presents evidence regarding the implementation of reusable materials and equipment, whereas support for other interventions is limited [8]. For this reason, it is necessary to conduct additional experiments that generate evidence for less studied interventions, such as waste separation or energy reduction strategies. Research must be oriented towards longitudinal studies that evaluate the long-term impact of sustainable interventions, considering the rapid evolution of energy sources. In addition, by developing appropriate software, the use of the method would be simplified while ensuring good traceability. We plan to undertake a multicenter validation study involving orthopedic departments across diverse hospital types and regions. This expanded study will incorporate quantitative reliability analyses, including Intraclass Correlation Coefficient (ICC) and Cohen’s kappa, to assess inter-assessor concordance. The goal will be to refine the indicator definitions, test applicability in different operational contexts, and enhance the robustness and transferability of the sustainability assurance framework. While our current approach to assessing indicator importance was based on expert consensus, we recognize the need for more formal statistical validation in future phases. The introduction of quantitative methods, such as ICC or Cohen’s kappa, for inter-rater reliability will provide greater methodological rigor. These methods will help distinguish between variability due to subjective interpretation and genuine differences in implementation across departments or institutions. Incorporating these measures will also strengthen the credibility and scalability of the proposed evaluation tool.

## 5. Conclusions

Sustainability assurance in orthopedic surgery is realized in four key domains: rationalization and reduction of consumption, correct separation and recycling of waste, reuse of textiles and equipment, and anesthetics and alternative interventions. Their continuous evaluation and improvement are achieved with the support of performance indicators, which are evaluated on scales with levels between 1 and 5. To this is added the evaluation of the indicators’ importance, similarly with values between 1 and 5. This evaluation methodology allows the use of a prioritization framework for improvement measures.

The four domains validated in practice at the orthopedic emergency hospital confirmed their suitability for the intended purpose but also their compatibility with other reference frameworks implemented in the hospital. Analysis of the theoretical effectiveness of sustainable interventions needs to be followed by showing how they can be widely implemented in real clinical practice. In this way, with the support of the value of the changes by the interested parties, surgeons, managers, and patients, their results will be long-lasting and more effective. Through this, the interested parties and the hospital will be increasingly oriented towards sustainability.

## Figures and Tables

**Table 1:** Sustainable orthopedic indicator matrix.

Level of Indicator → Indicator ↓	Level 1	Level 2	Level 3	Level 4	Level 5
Rationalization and reduction of consumption (I1)	Surgical washing	Energy consumption	Custom surgical trays	Sterilization	Reprocessing
Proper separation and recycling of waste (I2)	Information and education	Information system	Compliance with protocols	Treatment and sterilization	Recycling
Reuse of textiles and equipment (I3)	Reusable textiles	Reusable instruments in the emergency room and anesthesia	Reusable tools in interventions	Hybrid instruments	Reprocessing and ecological identification
Alternative interventions and anesthesia (I4)	Products with biopolymers	Anesthesia with ciprofol	Air pads	Intravenous anesthesia	Spinal anesthesia

**Table 2:** Assessment tool for sustainability assurance in orthopedic surgery.

No.	Indicator Descriptive	Level (Li)	Importance (Ii)	Sustainability Indicator (Si = Li·Ii)
1	(I1) Rationalization and reduction of consumption	4	3	12
2	(I2) Correct separation and recycling of waste	2	4	8
3	(I3) Reuse of textiles and equipment	3	2	6
4	(I4) Anesthetics and alternative interventions	4	5	20

Ii−Importance, Li−Level, Si−Sustainability Indicator.

## Data Availability

The data used in this study can be requested from the corresponding author.

## Abbreviations

The following abbreviations are used in this manuscript:

Si	Sustainability indicator
Li	Level
Ii	Importance
GSOSD	Global sustainability indicator
GSmaxOSD	Maximum global sustainability value for sustainable orthopedic surgery
FDRSOD	Fulfillment degree of the requirements related to sustainable orthopedic surgery
I1	Rationalization and reduction of consumption
I2	Proper separation and recycling of waste
I3	Reuse of textiles and equipment
I4	Alternative interventions and anesthesia
WALANT	Wide Awake Local Anesthesia No Tourniquet
ROI	Return on Investment
FMEA	Failure Modes and Effects Analysis
ICC	Intraclass Correlation Coefficient
ECHTM	Ethics Committee of the Targu Mures County Emergency Hospital

## Appendix A

**Table A1:** The indicator (I1)—Rationalization and reduction of consumption.

Indicator Level	Name of the Indicator Level	Indicator Level Description
1	Surgical wash	Disposable foam pads are replaced with reusable gel pads. Surgical handwashing is done with alcohol-based products that reduce the use of soap and water.
2	Energy consumption	Energy consumption is reduced by turning off workstations and operating room lights when not in use, using LED lighting and motion sensors. “Power Down” initiative where all lights are turned off and all equipment not used for anesthesia is turned off.
3	Customized surgical trays	Reduced surgical trays are used, on which instruments are placed that are used in more than 50% weight. It is used to custom “minimal” package of disposable surgical supplies. The size of the packaging of the customized surgical kits is appropriate.
4	Sterilization	Steam sterilization and packaging of reusable surgical instruments takes place in reusable rigid containers. When sterilizing and handling surgical equipment, the polypropylene plastic film is replaced by ubiquitous metal casings. The surgical principles of “minor field sterility” and Wide Awake Local Anesthesia No Tourniquet (WALANT) is applied.
5	Reprocessing	The electrical batteries in the operating room are redistributed. They are reprocessed and reused for external fixators, razors, blades, burs and tourniquets.

**Table A2:** The indicator (I2)—Proper separation and recycling of waste.

Indicator Level	Name of the Indicator Level	Indicator Level Description
1	Information and training	The national guidelines regarding the practice of medical waste management are aware of the health personnel. Provide training to sanitary staff, instructional posters in the work area regarding the continuous enforcement of waste management rules and regulations.
2	Compliance with protocols	Proper waste separation and recycling protocols are strictly followed. Color-coded baskets are provided.
3	Information system	A scoring system is used to improve biomedical waste segregation compliance and areas with low scores are regularly trained. Using Nightingale charts within the information management system, errors in waste segregation are identified and corrected.
4	Treatment and sterilization	New treatment solutions are used for the infusion bypass circuit by adding 2000 mL priming solution to the bypass circuit which is flushed completely and becomes clear. The infusion bypass circuit is diverted to the waste bag. The infusion circuit is sterilized, dechlorinated and recycled by polyvinyl chloride and polycarbonate.
5	Recycling	In the selection of reuse methods, the preservation of the functionality and original properties of the materials are considered. The recycling potential of arthroplasties is harnessed.

**Table A3:** The indicator (I3)—Reuse of textiles and equipment.

Indicator Level	Name of the Indicator Level	Indicator Level Description
1	Reusable textiles	Reusable face masks are used. Reusable laryngeal masks are used. Reusable surgical gowns are used.
2	Reusable instruments in emergency and anesthesia	Reusable pulse oximeters are used in emergency care. Reusable instruments are used in anesthesia.
3	Reusable tools in interventions	Reusable medical devices are used, such as laparoscopic instruments, laryngoscopes, and trocars. Reusable central venous catheter insertion kits are used. In single-level lumbar fusion surgeries, reusable instruments are used.
4	Hybrid instruments	Hybrid surgical instruments containing both disposable and reusable components are used. Robotic surgery uses hybrid products that are pre-dominantly reusable. There is only a small disposable component. Alternative surgical approaches using reusable equipment are preferred over minimally invasive surgery.
5	Ecological reprocessing and identification	Intermittent pneumatic compression sleeves are re-processed. Medical devices have eco-key numbers.

**Table A4:** The indicator (I4)—Alternative interventions and anesthesia.

Indicator Level	Name of the Indicator Level	Indicator Level Description
1	Products with biopolymers	Medical products incorporating biopolymers are used in cases requiring reduction of carcinogenic impact, non-cancerous impact and respiratory effects.
2	Anesthesia with ciprofol	Ciprofol is used only in cases where a high effectiveness of anesthesia is required.
3	Air pads	Instead of fluorinated gases, the use of an air buffer is preferred.
4	Intravenous anesthesia	Low-flow anesthesia and total intravenous anesthesia are used when spinal anesthesia cannot be used.
5	Spinal anesthesia	Spinal anesthesia is preferred.
